# Parkinson's Disease Risk and Alcohol Intake: A Systematic Review and Dose-Response Meta-Analysis of Prospective Studies

**DOI:** 10.3389/fnut.2021.709846

**Published:** 2021-10-14

**Authors:** Chuan Shao, Xiaoya Wang, Pan Wang, Hui Tang, Jiaquan He, Nan Wu

**Affiliations:** ^1^Department of Neurosurgery, Nanchong Central Hospital, The Second Clinical Medical College, North Sichuan Medical College, Nanchong, China; ^2^Department of Neurosurgery, Chongqing General Hospital, University of Chinese Academy of Sciences, Chongqing, China; ^3^Chongqing Medical University, Chongqing, China

**Keywords:** Parkinson's disease, risk factors, alcohol, ethanol, meta-analysis

## Abstract

**Background:** The association between Parkinson's disease (PD) risk and alcohol intake is a controversial topic.

**Objectives:** To systematically assess the association between PD risk and alcohol intake.

**Methods:** PubMed and Embase databases were searched for eligible studies with prospective design on PD risk and alcohol intake. A meta-analysis with a random-effects model and dose-response analysis was performed. Relative risk ratios (RRs) with 95% CIs were calculated.

**Results:** Eleven prospective studies were included. Overall, a higher intake of alcohol was inversely associated with PD risk (RR: 0.81, 95% CI: 0.70–0.95, *I*^2^ = 73.7%). Significant differences existed between the specific types of alcoholic beverages and geographic area. Specifically, a significant association existed for beer (RR: 0.78, 95% CI: 0.65–0.94, *I*^2^ = 0.0%) and studies conducted in Asia (RR: 0.66, 95% CI: 0.55–0.80, *I*^2^ = 37.3%). Dose-response analysis indicated a nonlinear relationship between PD risk and alcohol exposure. No evidence for publication bias was detected.

**Conclusions:** In summary, our meta-analysis suggests that alcohol consumption was associated with a decreased risk of PD, with a nearly U-shaped association. Future studies are warranted to clarify the question of a specific type of alcoholic beverage-dependent association, geographic area effect, and possible threshold effects regarding both the adverse and beneficial effects of alcohol.

## Introduction

Parkinson's disease (PD) characterized by three cardinal motor impairments: bradykinesia, rigidity, and tremor, is the second most common neurodegenerative disorder after Alzheimer's disease (1, 2). The incidence of PD increases with age and ~1% of the population > 60 years is affected by PD (2). Progress in understanding the etiology of PD is limited. Based on epidemiological studies, the broadest consistent evidence suggests that an inverse association between PD risk and cigarette smoking exists (3). Other risk factors, demonstrated with some consistency, include less physical activity, fewer doses of coffee intake, pesticides/insecticides, and increased intake of dairy products (4).

Alcohol consumption is another important lifestyle exposure following cigarette smoking and coffee consumption and is associated with multiple health outcomes, namely, cancers, neuropsychiatric disorders, and cardiovascular diseases (5, 6). Some evidence has shown that light to moderate alcohol intake has a decreased risk, while heavy intake carries an increased risk of disease or death (5, 6).

The brain is a key target of alcohol action. Prolonged and excessive alcohol consumption contributes to elevated oxidative stress, neuroimmune response, glutamate excitotoxicity, and their interplay effects that lead to permanent neuronal damage *via* mitochondrial lipid peroxidation and DNA or protein damage (7, 8). Thus, a potential association between alcohol intake and PD risk is hypothesized. The first study investigation was published in 1980 but did not yield significant findings (9). Since then, several studies have addressed the issue and both positive and negative results have been reported (10–30). Given the limitations of retrospective studies and single study power in previous studies, no strong evidence of an association exists. The aim of this systematic review and meta-analysis, therefore, was to quantitatively evaluate the effect of alcohol consumption on the risk of PD by summarizing the evidence from prospective studies using a dose-response approach.

## Methods

### Reporting Guideline

Our study was reported according to the Preferred Reporting Items for Systematic Reviews and Meta-Analyses statement (31).

### Literature Search

We identified all studies in the PubMed and EMBASE databases (up to August 21, 2021), and reference lists of relevant original papers, meta-analyses, and review articles. The following search terms were used: “{Parkinson[Title/Abstract] AND (liquor[Title/Abstract] OR ethanol[Title/Abstract] OR spirits[Title/Abstract] OR beer[Title/Abstract] OR wine[Title/Abstract] OR alcohol[Title/Abstract])}”. There were no publication years or language restrictions, but the search was limited to human studies.

### Selection Criteria

We included prospective cohort studies or case-control nested in prospective cohort studies that reported an association between alcohol consumption and PD risk and provided adjusted relative risks (RRs) and 95% CIs, or other data to estimate the association and risk. If multiple articles were based on the same case series, only the study with the longest follow-up period or largest sample size was included.

### Data Extraction

The last name of the first author, publication year, country, sample size, identification of PD, exposure variables of interest (wine, spirits/hard liquor, beer, and/or total alcohol), assessment of alcohol exposure, the adjusted RRs with corresponding 95% CIs in the multivariable analysis, and potential confounders considered were extracted from each eligible study.

### Statistical Analysis

Heterogeneity was measured using the *I*^2^ test (32). The random-effects model, incorporating the heterogeneity across studies, was adopted to combine the risk estimates (33). Subgroup analysis was performed according to gender, geographic area, and type of alcohol consumption. Sensitivity analysis was performed by excluding one study at a time. Another sensitivity analysis restricted to those studies that provided risk estimates adjusted for smoking and coffee intake was also performed. Potential publication bias was assessed by Begg's funnel plots and Egger's test (34).

In this study, grams were used as the measure for the assessment of alcohol exposure and we defined one drink as 13 g of ethanol (35), except when the dose was well-defined in the original study. We first performed a comparison of the highest vs. lowest level of alcohol exposure. A dose-response analysis with a one-stage robust error meta-regression (REMR) model (36) was adopted. Using the REMR model, only the study-specific adjusted RRs with 95% CIs for at least two levels of alcohol consumption were reported. When the median level of exposure range of alcohol exposure was unavailable, the midpoint of each category of alcohol consumption was used. When the upper exposure categories were open-ended, we assumed that the width was the same interval as the adjacent category. All analyses were performed using STATA software (version 15.0, STATA Corp., College Station, TX, USA).

## Results

### Literature Search

**Figure 1:**
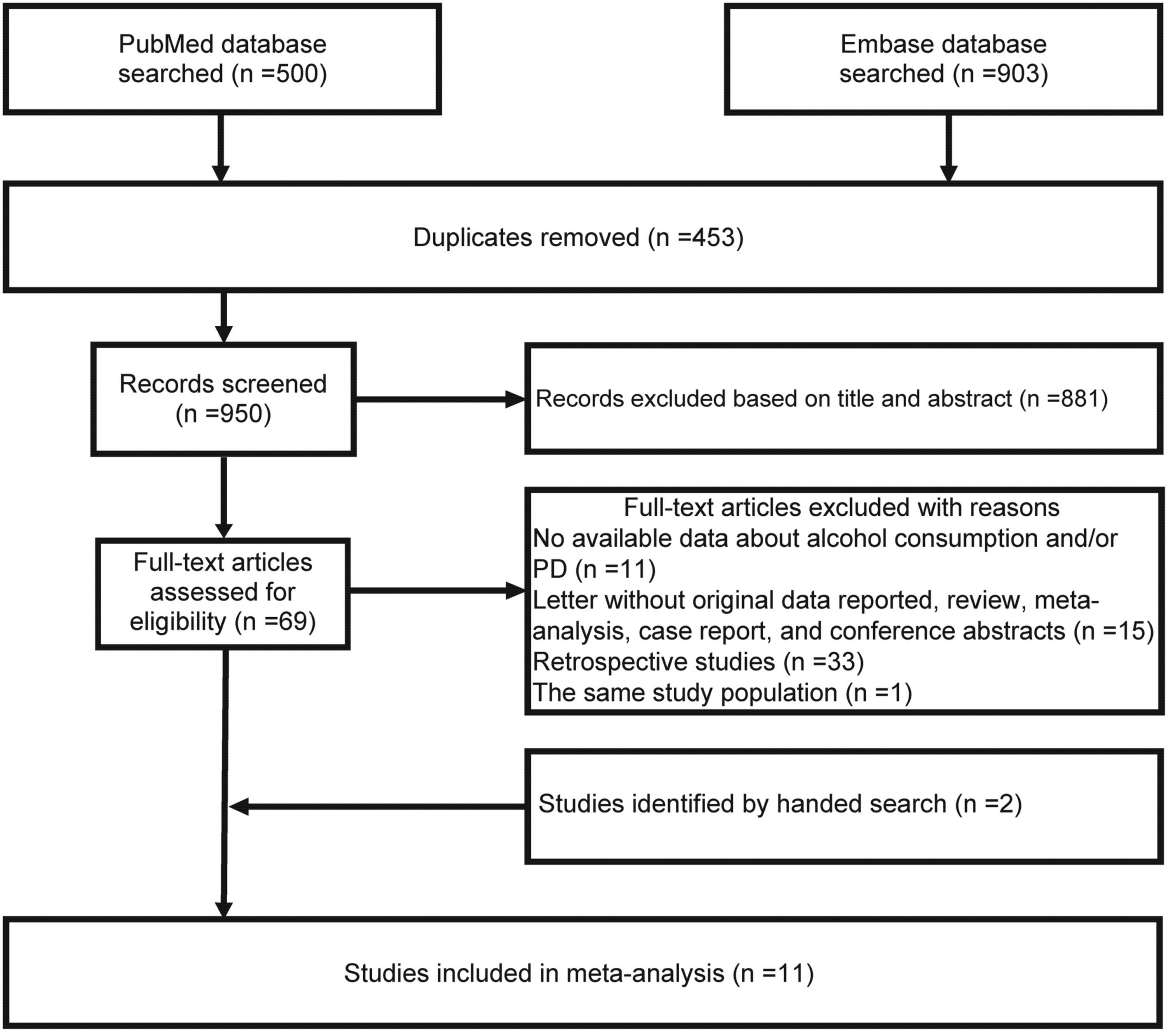
Flowchart for the article selection process.

Figure 1 shows the flowchart. The initial search of the PubMed and Embase databases identified 1,403 studies and 453 duplicate records were excluded. After examining the title and abstract, 881 studies were also removed, and thus, 69 studies remained for assessment of the full text. Sixty of 69 studies were further excluded for the following reasons: retrospective case-control studies, comment letters without original data reported, review, conference abstracts, case report, meta-analysis, reports of the same study population, and no data available regarding alcohol consumption and PD risk. The manual literature search identified two studies (22, 24). Thus, 11 eligible studies were included (20–30). We only included the data from external control subjects for the Swedish Twin Registry study in which two control groups were reported (23).

### Study Characteristics

Table 1 presents the basic characteristics of the included studies. There were three case-control studies nested within prospective cohorts (20, 22, 23) and eight prospective cohort studies (21, 24–30). Four studies were conducted in the United States (20, 21, 25, 26), two in Asia (24, 28), and five in Europe (22, 23, 27, 29, 30). Ascertainment of PD was identified by reviewing death certificates, hospital discharge summaries, and other types of medical records. All of the included studies had data on total alcohol intake, while only six studies had information on specific types of alcohol consumption (20, 21, 25–27, 29, 30). Alcohol exposure was measured using a mailed or self-administered questionnaire, or an in-person interview questionnaire. The reference category of alcohol exposure was nondrinkers in seven studies (20–23, 25–28) and light consumers (0–4.9 g/day) in another three studies (24, 29, 30).

**Table 1:** Characteristics of the included studies.

**Study**	**Country**	**Case/control (Cohort)**	**Case diagnosis**	**Exposure variables**	**Exposure assessment**	**Matching or adjustments**
Paganini-Hill (20)	USA	395/2,320	Death certificates and hospital discharge	Total alcohol (wine beer, hard liquor)	Mailed questionnaire	Sex, birth date, vital status, smoking, blood pressure medication, coffee, dietary vitamin C, and total vitamin A
Hernán et al. (21)	USA	415/136,089	Diagnosis by neurologist and medical record	Total alcohol (wine beer, hard liquor)	Mailed questionnaire	Age, smoking, and caffeine intake
Hernán et al. (22)	UK	1,019/10,123	Medical records	Total alcohol	Self-administered questionnaire	Age, sex, start date, and smoking
Wirdefeldt et al. (23)	Sweden	476/2,380	Death certificates and hospital discharge	Total alcohol	Mailed questionnaire	Sex, birth year, smoking, coffee intake, and educational level
Tan et al. (24)	Singapore	151/63,257	Hospital discharge	Total alcohol	Interview questionnaire	Age, sex, education, smoking, coffee, tea, dialect group, and year of interview
Palacios et al. (25)	USA	605/132,403	Diagnosis by neurologist and medical record	Total alcohol (wine beer, hard liquor)	Mailed questionnaire	Age, sex, race, education, marital status, smoking, caffeine intake, physical activity, and self-evaluated health status
Liu et al. (26)	USA	1,113/306,895	Medical records	Total alcohol (wine beer, hard liquor)	Self-administered questionnaire	Age, sex, race, education, marital status, smoking, caffeine intake, physical activity, and self-evaluated health status
Sääksjärvi et al. (27)	Finland	101/6,715	Nationwide register	Total alcohol	Self-administered questionnaire	Age, sex, education, community density, occupation, coffee consumption, smoking, alcohol consumption, BMI, and leisure-time physical activity
Kim et al. (28)	Korea	28041/6,795,816	Medical records	Total alcohol	Self-administered questionnaire	Age, regular exercise, body mass index, total cholesterol, fasting blood glucose, and the presence of diabetes mellitus, and smoking
Peters et al. (29)	European	694/209,998	Clinical record	Total alcohol (beer, wine, spirits)	Self-administered questionnaire	Age, sex, country, smoking status, and coffee consumption
Kim et al. (30)	UK	11,009/1,309,267	Inpatient or day-case hospital admission record and/or death registration	Total alcohol (wine beer, hard liquor)	Self-administered questionnaire	Age, smoking, region, deprivation index, educational attainment, strenuous exercise, body mass index, diabetes, hypertension, heart disease, stroke, and ever HRT use

*UK, United Kingdom; BMI, body mass index*.

### Main Analysis

**Figure 2:**
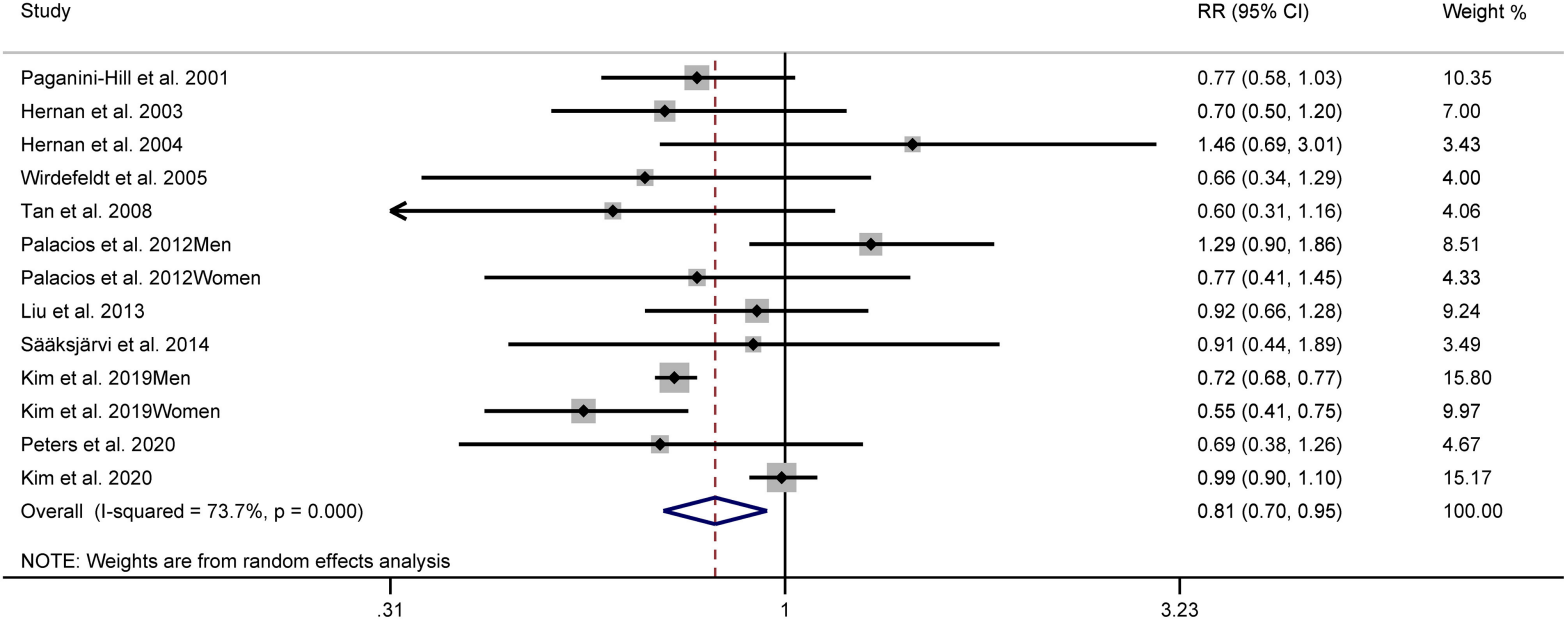
Forest plots for the relationship between Parkinson's disease (PD) risk and alcohol consumption.

Figure 2 shows the study-specific adjusted RRs with 95% CIs and pooled results. The overall analysis suggested that the highest category of alcohol consumption was associated with a decreased risk of PD (RR: 0.81, 95% CI: 0.70–0.95). Substantial heterogeneity was identified (*I*^2^ = 73.7%).

Significant differences existed based on stratified analyses by geographic area. The pooled RRs were 0.66 (95% CI: 0.55–0.80, *I*^2^ = 37.3%), 0.88 (95% CI: 0.71–1.10, *I*^2^ = 37.3%), and 0.98 (95% CI: 0.89–1.08, *I*^2^ = 0.0%) for Asian, American, and European studies, respectively. No significant association between alcohol consumption and PD risk was demonstrated when a stratified analysis was based on sex. The pooled RRs were 0.81 (95% CI: 0.61–1.08, *I*^2^ = 61.3%) in male studies and 0.79 (95% CI: 0.57–1.08, *I*^2^ = 64.0%) in female studies. Considering subgroups of specific types of alcoholic beverages, the pooled results showed a RR of 0.78 (95% CI: 0.65–0.94, *I*^2^ = 0.0%) for beer, 0.98 (95% CI: 0.77–1.24, *I*^2^ = 58.0%) for liquor, and 0.95 (95% CI: 0.85–1.08, *I*^2^ = 2.5%) for wine.

Sensitivity analyses suggested that our results were not stable. Exclusion of the national program of 6,795,816 Koreans aged ≥ 40 years (24) did not yield a significant association (RR: 0.90, 95% CI: 0.79–1.03). Furthermore, heterogeneity was markedly decreased (*I*^2^ = 24.1%).

Another sensitivity analysis was restricted to those studies that provided risk estimates adjusted for smoking and coffee (20, 21, 23–27, 29). The pooled RR was 0.84 (95% CI: 0.72–0.98, *I*^2^ = 8.1%).

**Figure 3:**
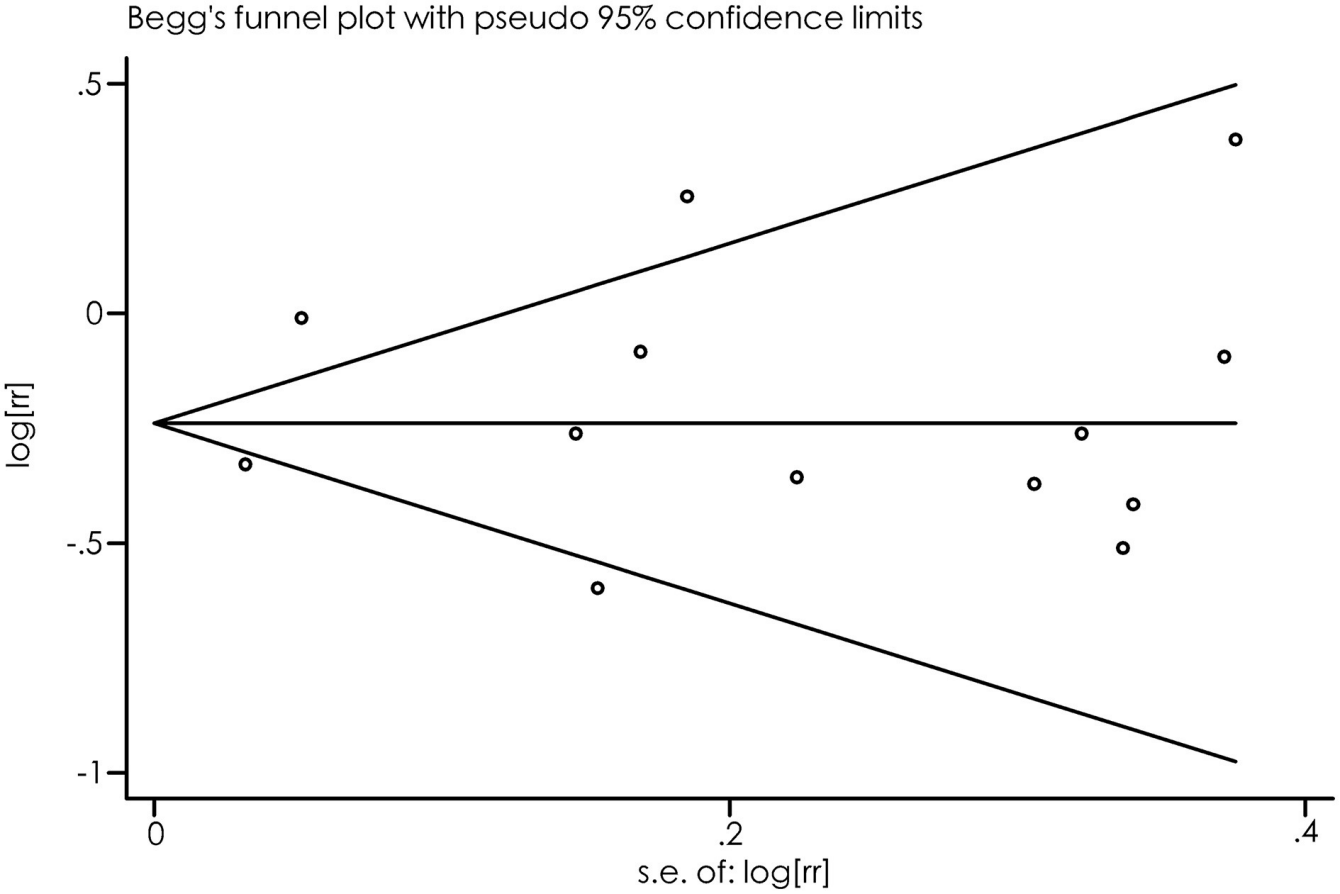
Begg's funnel plot for the relationship between PD risk and alcohol consumption.

As suggested by Begg's funnel plots (Figure 3) and Egger's linear regression test (*p* = 0.697), no evidence of publication bias was found.

**Figure 4:**
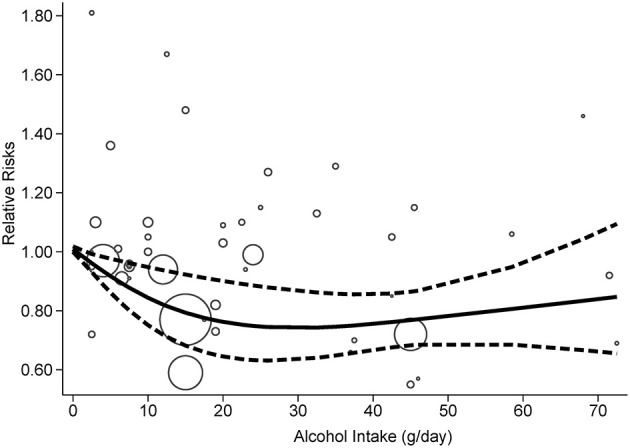
Dose-response relationships between PD risk and alcohol consumption.

According to the criteria of the REMR model, 10 studies were included in the dose-response analysis (20–23, 25–30). A nonlinear association (*p* = 0.0018) between alcohol consumption and the risk of PD was confirmed (Figure 4). The strongest inverse association was observed for consumption of 26–35 g/day and no further reduction in risk of PD with increasing consumption of alcohol over this mount was identified.

## Discussion

In this comprehensive meta-analysis of 11 prospective studies, we found a slightly decreased risk of PD in those who consume alcohol. Dose-response analysis provided evidence for a significant, nonlinear, and inverse association between PD risk and alcohol consumption.

Interestingly, previous evidence hinted that there may be significant differences in the risk of PD associated with specific types of alcoholic beverages (20, 21, 25, 26, 29, 30). Two studies showed that beer consumption was associated with a decreased risk of PD, but wine or liquor with an increased risk of PD, although some results were not statistically significant (21, 26). In another four studies, there was no difference between beer, wine, and liquor, with a non-significant inverse (20, 29, 30) or positive (26) relationship reported. After pooling these results, we found that a decreased PD risk was only associated with beer consumption, but not liquor and wine. This indicated that other ingredients except ethanol may confer a beverage-specific relationship. Beer has a much lower ethanol content, but higher antioxidants levels, such as folic acid, niacin, purine, and other phenolics, which were thought to mediate the neuroprotective effects of alcohol (7, 37). Moreover, beer beverage intake is associated with high urate concentration, which has been shown to be neuroprotective effects in animal models (7, 38). There is also limited epidemiological evidence that uric acid (UA) prevents the development of PD. In a Netherlands cohort of approximately 5,000 participants (age ≥ 55 years; the average follow-up, 9.4 years), De Lau et al. (39) found that a higher concentration of UA was independently associated with a lower risk of PD, with significant evidence for a dose-exposure relationship (39). Another US cohort of 15,792 participants aged 45–64 years with nearly 20 years of follow-up also supported the previous finding that UA may be a protective factor against PD, especially in men (40). Taken together, it is thought that beer consumption may be associated with a deceased PD risk; however, this issue warrants further verification in future studies.

Our stratified analyses based on gender indicated that no significant association existed in women or men. Further analysis by geographic area revealed a significant association for Asia, but not the United States or Europe. The significance of geographic area disparities is unclear. One possible explanation is that a chance result was obtained in the US or European group as the limited number of included studies were included. An alternative explanation was that the potential residual confounding effect could not be excluded in the Asia group. The Korean study revealed a strong effect (RR range, 0.55–0.77) (28), but the risk estimates were not adjusted for caffeine intake, an important risk factor for PD. Finally, different lifestyles and genetic backgrounds may account for part of the difference.

In the dose-response analysis, a nearly U-shaped association between PD risk and alcohol consumption was identified, with the strongest inverse association observed for consumption of 26–35 g/day and no further reduction in risk of PD with increasing consumption of alcohol over this amount. Similarly, a 2019 systematic review of meta-analyses covering 34 risk factors concluded that light or moderate alcohol consumption was associated with a decreased risk of Alzheimer's disease (41). Regarding the cognitive decline, there was some evidence that the relationship with alcohol consumption was thought to be U- and J-shaped (8). All of these findings were supported by current biological mechanisms of alcohol conferring neurodegenerative disorders risk, with a protective or harmful effect reported in several reviews (7, 8, 42). A wide range of dose-risk estimates was identified in our study; however, additional studies are needed to provide a precise estimate.

Several meta-analyses of the relationship between alcohol consumption and PD risk have been published (35, 43–45). In a meta-analysis of 13 case-control studies published until September 2004, Ishihara and Bryne reported that ever alcohol drinkers were associated with a decreased risk of PD compared with nondrinkers (RR: 0.81, 95% CI: 0.70–0.92) (43). In 2012, another meta-analysis of 22 case-control and two cohort studies assessed alcohol exposure as “drinking” vs. “nondrinking” and the result (RR: 0.90, 95% CI: 0.84–0.96) (44) were in agreement with Ishihara and Bryne (43). Two years later, a meta-analysis of 24 case-control studies and eight prospective studies showed that alcohol consumption was inversely associated with PD risk (35). Furthermore, the dose-response curve showed a significant negative, linear association (35). In 2019, based on 26 case-control and five prospective studies, Jiménez-Jiménez et al. (45) estimated crude and diagnostic odds ratios with 95% CIs to measure the association between PD risk and alcohol consumption. An inverse association between PD risk and alcohol consumption was only supported by evidence of retrospective case-control studies but not prospective ones (45). Noticeable, in previous meta-analyses, the vast majority of included studies were retrospective studies that were always subjected to recall and selective biases. Additionally, we performed a comparison of the highest vs. lowest categories of alcohol exposure rather than “ever” or “never,” which is thought to be inadequate to assess the complexity of alcohol (46). Thus, our meta-analysis of 11 prospective studies may provide a relatively precise estimation. Specially, a nonlinear relationship between PD risk and alcohol exposure was observed.

The main strengths of this meta-analysis included a large number of prospective studies with large sample sizes, estimation of total and specific types of alcoholic beverages, and dose-response analysis with the one-stage method. Also, some potential limitations need to be addressed. First, significant heterogeneity was identified. As indicated by the results of sensitivity and subgroup analyses, confounding factors may have accounted for, in part, heterogeneity. Second, because of the nature of observational design, a residual confounding effect could not be neglected. Third, we noted that the potential measurement bias of alcohol exposure may twist the true relationship between alcohol consumption and PD risk. For example, alcohol exposure was only measured in the past 12 months before baseline, but not lifetime (26), and thus, “former drinkers” may become “never drinkers” at the baseline examination and the relationship between alcohol exposure and PD risk may have been distorted. A further study providing a more detailed exposure, namely, “former drinkers” and “current drinkers” would help us fully understand the relationship between alcohol exposure and PD. Fourth, our results were not stable. When the Korean study (28) was removed from the sensitivity analyses, there was no significant association between PD risk and alcohol intake. Finally, potential publication bias is always a concern in meta-analyses, although little evidence for publication bias was identified by Begg's funnel plots and Egger's linear regression test.

In conclusion, our meta-analysis suggested that alcohol consumption was associated with a decreased risk of PD (a nearly U-shaped association). Due to the above-listed limitations, the need for caution regarding our findings is warranted. The specific type of alcoholic beverage-dependent association, geographic area effect, and possible alcohol threshold for adverse and beneficial effects remain to be clarified in future studies.

## Data Availability Statement

The original contributions presented in the study are included in the article/supplementary material, further inquiries can be directed to the corresponding author/s.

## Author Contributions

CS and HT designed the research and wrote the first draft. CS and XW performed the second literature search. CS, HT, XW, and PW extracted and confirmed the data. CS performed the statistical analysis. CS, HT, XW, PW, NW, and JH reviewed and rewrote the study. All authors contributed to the article and approved the submitted version.

## Conflict of Interest

The authors declare that the research was conducted in the absence of any commercial or financial relationships that could be construed as a potential conflict of interest.

## Publisher's Note

All claims expressed in this article are solely those of the authors and do not necessarily represent those of their affiliated organizations, or those of the publisher, the editors and the reviewers. Any product that may be evaluated in this article, or claim that may be made by its manufacturer, is not guaranteed or endorsed by the publisher.
